# Estimations of Critical Clear Corneal Incisions Required for Lens Insertion in Cataract Surgery: A Mathematical Aspect

**DOI:** 10.3389/fphys.2022.834214

**Published:** 2022-04-08

**Authors:** Nan Qi, David Lockington, Lei Wang, Kanna Ramaesh, Xiaoyu Luo

**Affiliations:** ^1^ Institute of Marine Science and Technology, Shandong University, Qingdao, China; ^2^ Tennent Institute of Ophthalmology, Gartnavel General Hospital, Glasgow, United Kingdom; ^3^ Beijing National Center for Applied Mathematics, Academy for Multidisciplinary Studies, Capital Normal University Beijing, Beijing, China; ^4^ School of Mathematics and Statistics, University of Glasgow, Glasgow, United Kingdom

**Keywords:** intra-ocular lens (IOL) injection system, tissue damage, energy release rate, linear fracture mechanics, finite element analysis

## Abstract

In a routine cataract operation cornea tissue may be damaged when an intra-ocular lens (IOL) injector of diameter between 1.467 and 2.011 mm is inserted through an empirically designed 2.2 mm corneal incision. We aimed to model and estimate the minimal length of the incision required to avoid wound tear. It was assumed that the damage was caused by tissue fracture at the tips of the incision, and this fracture could be studied using damage and fracture mechanics. The criterion of the damage was caused by a tear governed by the critical energy release rate (ERR) *G*_*c*_, which is tissue dependent. Analytical and numerical studies were both conducted indicating the possibility of a safe and effective incision in cataract surgery. Six commonly used IOL injection systems were examined. Our results suggested that the recommended 2.2 mm incision cannot be treated as a universal threshold. Quicker IOL insertion may reduce wound damage. It was also recommended to advance IOL injector *via* its minor axis, and to cut the tear preferably along the circumferential direction due to tissue orthotropy. This study provides useful information and a deeper insight into the potential for mechanical damage to the corneal wound in cataract surgery.

## 1 Introduction

Advances in ocular surgical techniques have resulted in significant reductions in the dimensions of the surgical incisions required to extract the cataractous lens and replace it with an intra-ocular lens (IOL). These smaller wound developments have clinically contributed to safer surgery, with quicker procedures, faster recovery and minimal surgically induced astigmatism (Al Mahmood et al., 2014; Bernhisel and Pettey, 2020). The advent of foldable IOLs further encouraged smaller wound sizes. The most common size of IOL optic is a 6 mm diameter circle, with two legs (haptics) resulting in an approximate IOL length of 12 mm (Randleman and Lockwood, 2016). To deliver these foldable IOLs into eyes in a micro-incisional cataract surgery, an appropriate injector system (manual or pre-loaded) is required to permit safe insertion through a corneal wound (generally around 2.2 mm in length) (Nanavaty and Kubrak-Kisza, 2017; Kim et al., 2014; He et al., 2021; Zhang et al., 2022).

The manufacturers of these micro-incisional injector systems often claim that their device fits through a 2.2 mm incision, however, it is clinically common to experience significant tissue resistance during IOL delivery. To address this resistance, the surgeon has two options: either to twist and force the IOL through the wound (so called “wound-assisted”), or surgically widen it with a surgical keratome blade. Corneal incisions for intra-ocular surgery have long been associated with varying degrees of surgically induced astigmatism, so smaller incisions are preferable (Shepherd, 1989; Kim et al., 2014). However, the surgeon is trying to balance this against the potential for causing irregular forced tissue damage.

Previous studies have compared and investigated the relationship between various types of IOLs, injector systems, insertion techniques, insertion speeds and incision sizes (Ouchi, 2012; Kim et al., 2014; El Massry et al., 2016; Nanavaty and Kubrak-Kisza, 2017). Smaller incision sites may be more prone to corneal trauma, including microtears and tissue stretching (Matossian et al., 2015; Oshika and Wolfe, 2019). The internal wound stretch (the maximum post-IOL implantation wound size and percentage stretch) between 5 preloaded IOL systems and the implications on wound integrity have been previously investigated in porcine eyes (Nanavaty and Kubrak-Kisza, 2017).

Reliable wound construction is essential. If the corneal incision is inadequate in length and subjected to further stretching by surgical instrumentation or during forced insertion of the IOL, it is less likely to maintain its integrity, leak aqueous and be a potential reason for post-operative infection (El Massry et al., 2016). We wondered if a forced wound stretch is more likely to be incompetent due to tissue damage/fracture compared to a surgically sharp blade extended wound. We wished to identify the key factors affecting the wound stretch in order to make recommendations as to the optimum wound size for IOL insertion *via* commonly used IOL injectors.

In this study, a simplified analytical model is provided to investigate this clinical scenario, and its theory based on the concept of energy release rate in fracture mechanics is described, followed by a numerical finite element simulation, conducted in Section 2. In Section 3, results are presented with the dimensions of commonly used IOL injectors and followed by discussions and conclusions.

## 2 Methods

### 2.1 Energy Release Rate

To find an optimal incision size that is adequate in length to avoid tissue stretching along the incision line due to injection and as small as possible to reduce its impact on wound integrity, the priority is to correlate the tissue failure with IOL injector options.

To describe material failure in damage and fracture mechanics, one very commonly used mathematical theory is based on calculating the energy release rate (ERR), G, which was developed by, among others, Griffith (Griffith, 1921) and extended by Irwin and Wells (Irwin and Wells, 1965). It is the energy per unit length released from the system by extending the tear surface by an infinitesimal length d*a* in plane problem, i.e., *G* = −dΠ/d*a*, where *Π* is the mechanical energy. The concept of ERR stems from the energy balance principle during an infinitesimal quasi-static tear extension. The total potential energy of a sample of tissue with a tear under deformation can be expressed as

$$E=\Pi +G_{c}a,$$ (1)

where the mechanical energy *Π* = *Ψ* − *W*, *Ψ* is the total strain energy and *W* is work done by loads. *G*_*c*_, as a material parameter, is the critical energy required to break all atomic bonds per unit length in a two dimensional (2D) problem as studied here and *a* is the total tear length. The minimal potential energy principle requires that

$$\frac{\text{d}E}{\text{d}a}=\frac{\text{d}\Pi }{\text{d}a}+G_{c}<0,$$ (2)

which is equivalent to

$$G>G_{c}.$$ (3)

This is the criterion to determine if the tear may propagate (i.e., it is energetically feasible); otherwise, it is stationary.

### 2.2 Model Set-Up

By assuming the cornea be a simple linear material and ignoring its curvature, we considered a 2D infinite plate containing a pre-existing incision of length 2*a*, the incision size was assumed to be relatively small compared to the cornea studied.

**FIGURE 1:**
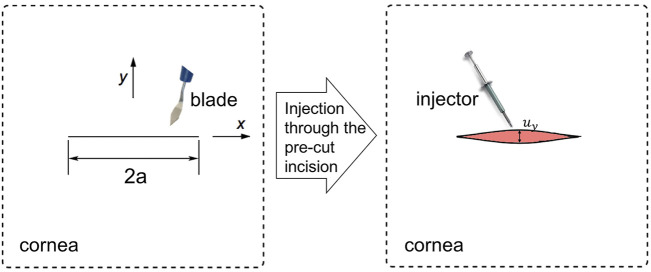
In a 2D infinitely large cornea tissue piece, a incision cut by a blade of length 2*a* was subjected to a injector, which expanded the tissue to an Mode I opening.

By applying an IOL injector through the incision, the line incision opened and expanded to a diamond shape, as shown in Figure 1. This was a basic fracture mode called Mode I or Opening Mode, i.e., the two crack surfaces experienced a jump only in *u*_*y*_, that is, they moved away symmetrically with respect to the undeformed crack plane.

It can be shown that the general displacement solution of the upper crack surface for the Mode I problems is given by (Westergaard, 1933; Sun et al., 2012) (see detailed derivations *via* Westergaard function in Supplementary Material)

$$u_{y}=\frac{\kappa +1}{4\mu }\frac{K_{I}}{\sqrt{\pi a}}\sqrt{a^{2}-x^{2}},$$ (4)

where $\mu =\frac{E}{2\left(1+\nu \right)}$ is the shear modulus, *E* and *ν* are the Young’s modulus and the Poisson’s ratio of cornea, *κ* = (3 − *ν*)/(1 + *ν*) in a plane stress problem. *K*_*I*_ is the stress intensity factor, which describes the state of stress at the edges, and depends on different boundary conditions.

To permit one injector to apply through, the displacement of the crack surface at *x* = 0 was at least the same size of half the injector’s smallest diameter, i.e., half of its minor axis *b*/2. Thus,

$$\frac{2K_{I}}{E}\sqrt{\frac{\pi }{a}}=\frac{b}{2}.$$ (5)

Using the relationship between ERR and the stress intensity factor $G=\frac{K_{I}^{2}}{E}$ and the criterion to keep a stationary tear in Eq. 3, the critical incision length 2*a*_*c*_ could then be obtained by

$$2a_{c}=\frac{\pi Eb^{2}}{8G_{c}}$$ (6)

It was clear to see that the allowed incision length was proportional to the injector size, decreases linearly with critical ERR, and increased linearly with the Young’s modulus.

### 2.3 Finite Element Analysis

**FIGURE 2:**
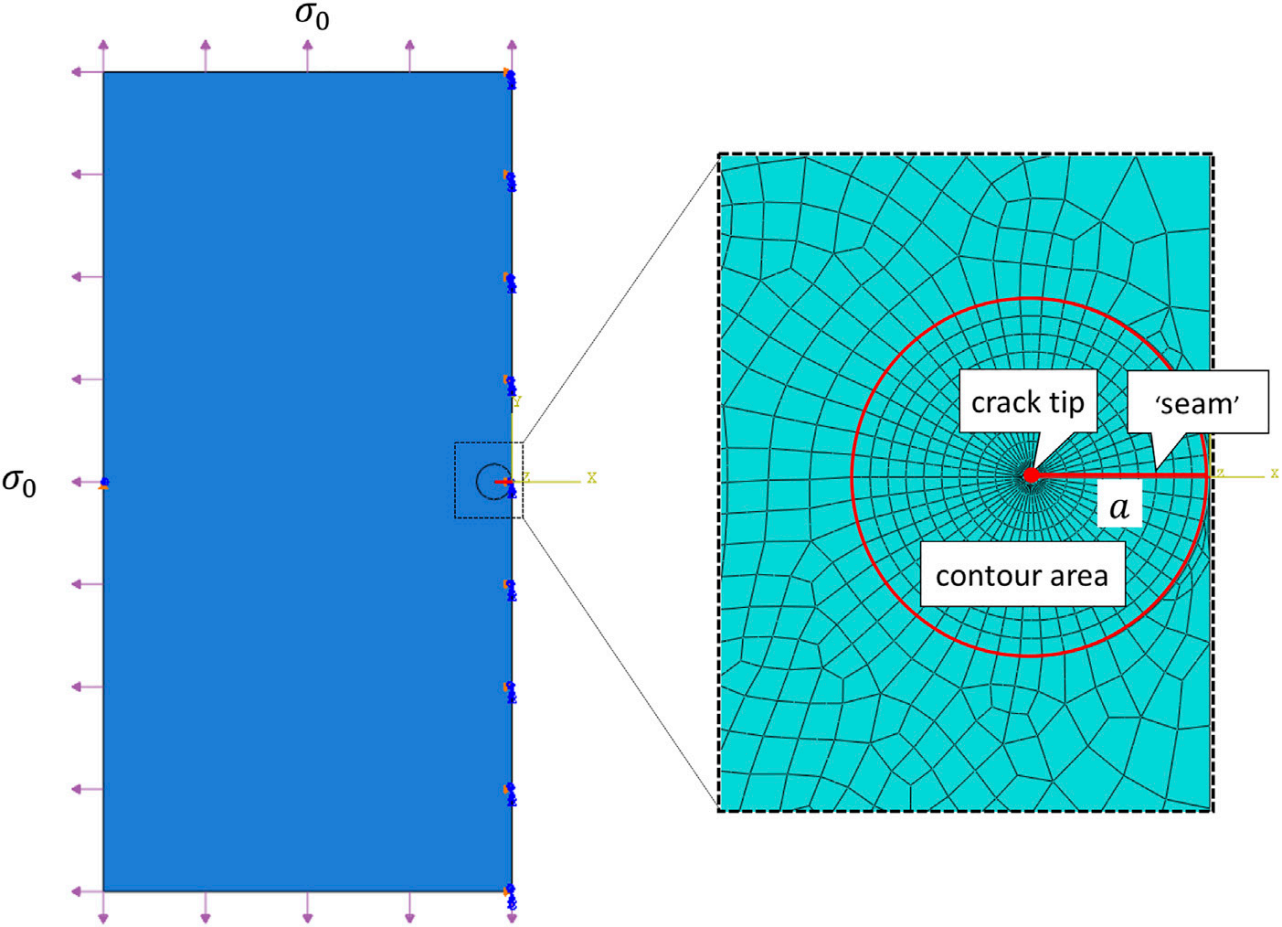
Sketch of a large 2D plate with a transverse tear of length 2*a* under a uniformly distributed stress *σ*_0_ at each edge. Note that only left half of the problem was conducted in the FE simulation. The insert shows the designed FE mesh generation, where the crack tip was surrounded by a sweeping contour area for ERR calculation.

Consider a large square sample (60 mm × 60 mm^1^) with a 2*a* mm crack in the middle, a classical Mode I boundary condition, i.e., a uniformly distributed stress *σ*_0_ at each edge, was applied. And the amount of stress being applied was adjusted so that the displacement of the crack surface in the middle is *b*/2. From analytical derivation $\sigma_{0}=\frac{Eb}{2a}$ and details analytical derivation could be found *via* Supplementary Equation S8 in Supplementary Material. Only left half of this symmetric problem was simulated in the finite element (FE) simulation and depicted in Figure 2. The right edge was fixed in *x* direction to secure symmetry, the central point on the left edge was all fixed to avoid rigid body motion.

The finite element simulations were performed using the commercially available finite element package Abaqus, 2013, and was conducted on Intel(R) Core(TM) i7-10700 CPU at2.90 GHz machine with 32.0 GB RAM. The crack was firstly represented by a partition and created by defining a “seam”, where nodes on elements on each side of the crack could be separated. ERR, as a contour energy integral, was calculated for layers of elements in rings, this required a spider web-like mesh generated around the crack tip. Sweeping strategy was selected and triangular element was wrapped around the crack tip. In particular, each ring of elements along the crack corresponded to a contour integral. For a typical simulation, we used a total of 23,881 nodes and 7,870 elements (CPS4R: Bilinear elements using reduced integration with hourglass control), and as shown in Figure 2, 10 contours were generated for ERR evaluations. One simulations took about 3–5 min with a reasonable initialization. The grid size was chosen following a grid independence test (simulations were run for increasingly refined grids until the results converged).

In FE simulations, Abaqus scripts were developed in Linux shell, so as to easily, automatically and robustly alternating the values of crack length *a* and communicating to its corresponding distributed stress *σ*_0_. This powerful tool allowed us to combine the functionality of the Graphical User Interface (GUI) of Abaqus and the power of the programming language *Python*.

## 3 Results

### 3.1 Analytical Results

Assuming that the cornea was isotropic, typical strain rate-dependent material parameters of *E* and *G*_*c*_ from porcine cornea (Tonsomboon et al., 2014) were listed in Table 1. Six preloaded IOL delivery system with different diameters were compared and listed in Table 2. These company products (Nanavaty and Kubrak-Kisza, 2017; Zhang et al., 2022) are Ultrasert (U) (Alcon Laboratories, Inc.), Eyecee (E) (Bausch & Lomb, Inc.), iSert (iS) (Hoya Surgical Optics, Inc.), CT Lucia (CT) (Carl Zeiss Meditec AG), iTec (iT) (Abbott Medical Optics, Inc.) and Rayone (R) (Rayner Intraocular Lenses Ltd.), respectively. All IOLs have a straw-like bevel face at the tip of its nozzle to aid delivery and their sizes in both axial and profile views are detailed in Table 3. The majority of injectors have a circular cross-section (e.g., R injector had major axis length of 1.739 mm and minor axis of 1.734 mm). Injectors U and E had a noticeable elliptical major-minor axial ratio of 1.37 and 1.14.

**TABLE 1:** Material parameters for healthy porcine corneas at different strain rates (Tonsomboon et al., 2014).

Strain rate (*mm* ⋅ min^−1^)	*E* (*MPa*)	*G*_*c*_ (*kJ*/*m* ^2^)
3 (slow)	9.59	3.39
30 (medium)	10.29	4.40
300 (fast)	9.82	5.40

**TABLE 2:** Critical incision lengths for six IOL injection systems at various incision speeds. The critical length larger than recommended 2.2 mm are shown in bold.

IOL injector	Minor axis *b* (mm)	Injection speed	Critical length 2*a*_*c*_ (mm)
U	1.467	Fast	1.537
		Medium	1.976
		Slow	**2.391**
E	1.671	Fast	1.994
		Medium	**2.564**
		Slow	**3.102**
R	1.734	Fast	2.147
		Medium	**2.761**
		Slow	**3.340**
iT	1.829	Fast	**2.389**
		Medium	**3.072**
		Slow	**3.716**
iS	1.834	Fast	**2.402**
		Medium	**3.089**
		Slow	**3.737**
CT	1.847	Fast	**2.436**
		Medium	**3.133**
		Slow	**3.790**

**TABLE 3:** The sizes of all IOLs in axial and profile views (Nanavaty and Kubrak-Kisza, 2017). The minor axis length (in bold) were selected for calculations.

IOL injector	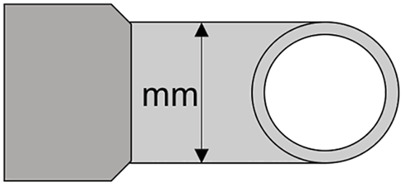	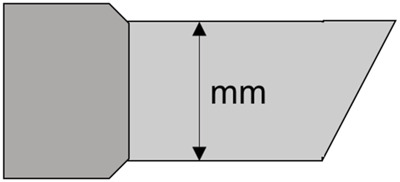
U	2.011	**1.467**
E	1.902	**1.671**
R	1.739	**1.734**
iT	**1.829**	1.847
iS	**1.834**	1.870
CT	**1.847**	1.852

**FIGURE 3:**
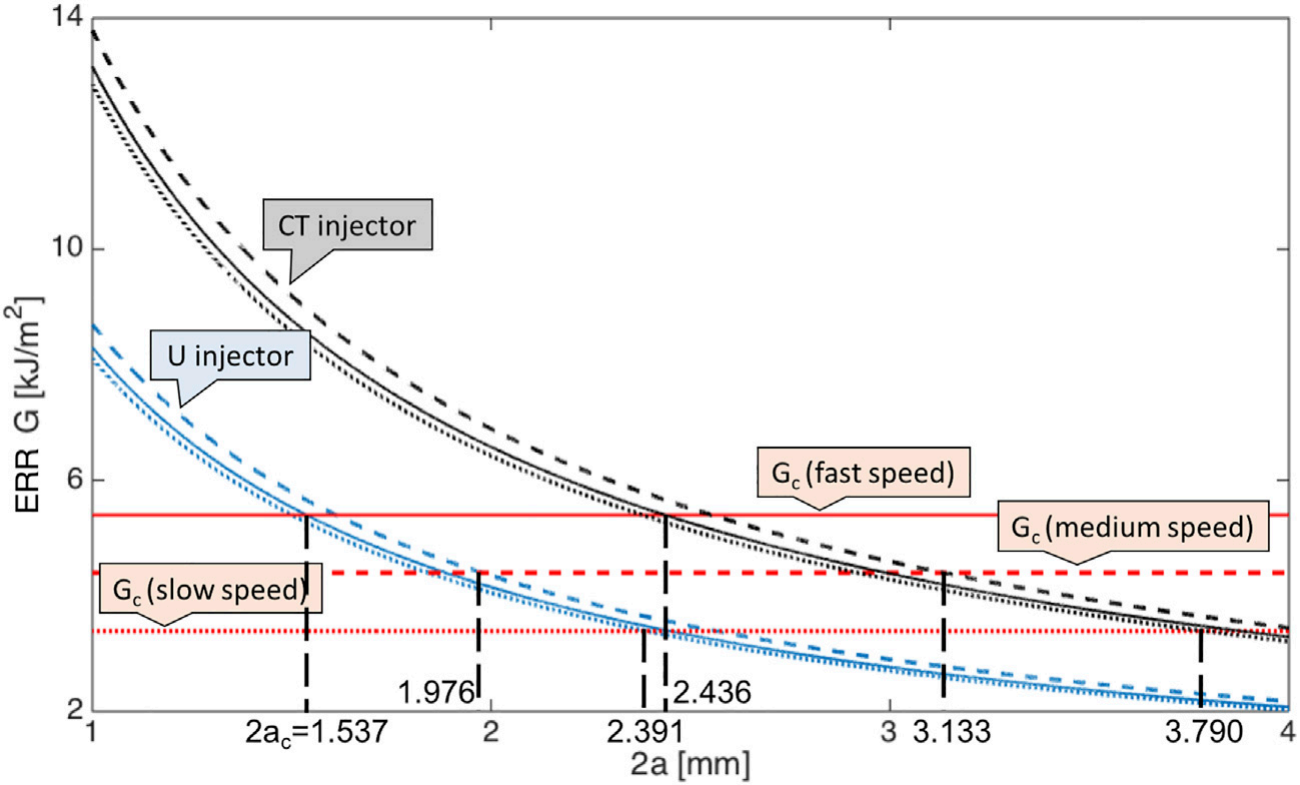
Energy release rates for an isotropic large plate plotted against the incision length 2*a* at three incision speeds given CT (black lines) and U injection systems (blue lines). Results at fast speed are in solid lines, medium speed in dashed lines and slow speed in dotted lines. The corresponding critical energy release rates *G*_*c*_ (red lines) are also given for reference. The six junctions points between same type of lines represent the critical lengths 2*a*_*c*_.

By applying Eq. 6, the corresponding energy release rate *G* against incision length 2*a* for two representative injection systems CT and U, i.e., with largest and smallest injector sizes, was depicted in Figure 3. As clearly presented in Eq. 6, with larger Young’s modulus at medium speed as listed in Table 1, the calculated ERR was bigger with same incision length. However, the difference of speed-dependence Young’s modulus was small compared to corresponding speed-dependent *G*_*c*_, so the critical 2*a*_*c*_ reaches first at fast speed which had largest *G*_*c*_, and further at medium and lastly at slow speeds. The pattern was valid for these two injectors and also for all injection system, as presented in Table 2, listing quantitative results for all six injectors. It is also be observed from Table 2 that the larger the injector size, the bigger the required safe incision length. The intersection of *G* and *G*_*c*_ lines gave the allowed minimal incision length, below this length, the pre-existing incision became unstable and torn. In particular, at fast incision speed, it was energetically stationary with a tear length 2*a* ≥ 1.537 mm for injector U delivery and 2*a* ≥ 2.436 mm for injector CT delivery. At medium incision speed, the incision was safe with a tear length 2*a* ≥ 1.976 mm and 2*a* ≥ 3.133 mm for U and CT advancement, respectively. The critical minimal tear length were 2.391 and 3.790 mm at slow speed.

Among all the given injectors (Nanavaty and Kubrak-Kisza, 2017), the minimum incision length was 1.537 mm for U system at fast incision speed, this value is below the recommended value of 2.2 mm (Nanavaty and Kubrak-Kisza, 2017; Kim et al., 2014), which indicates the operation can be conducted with no further damage. However, many other choices required larger pre-existing incision, and were highly dependent on the injection speed.

**FIGURE 4:**
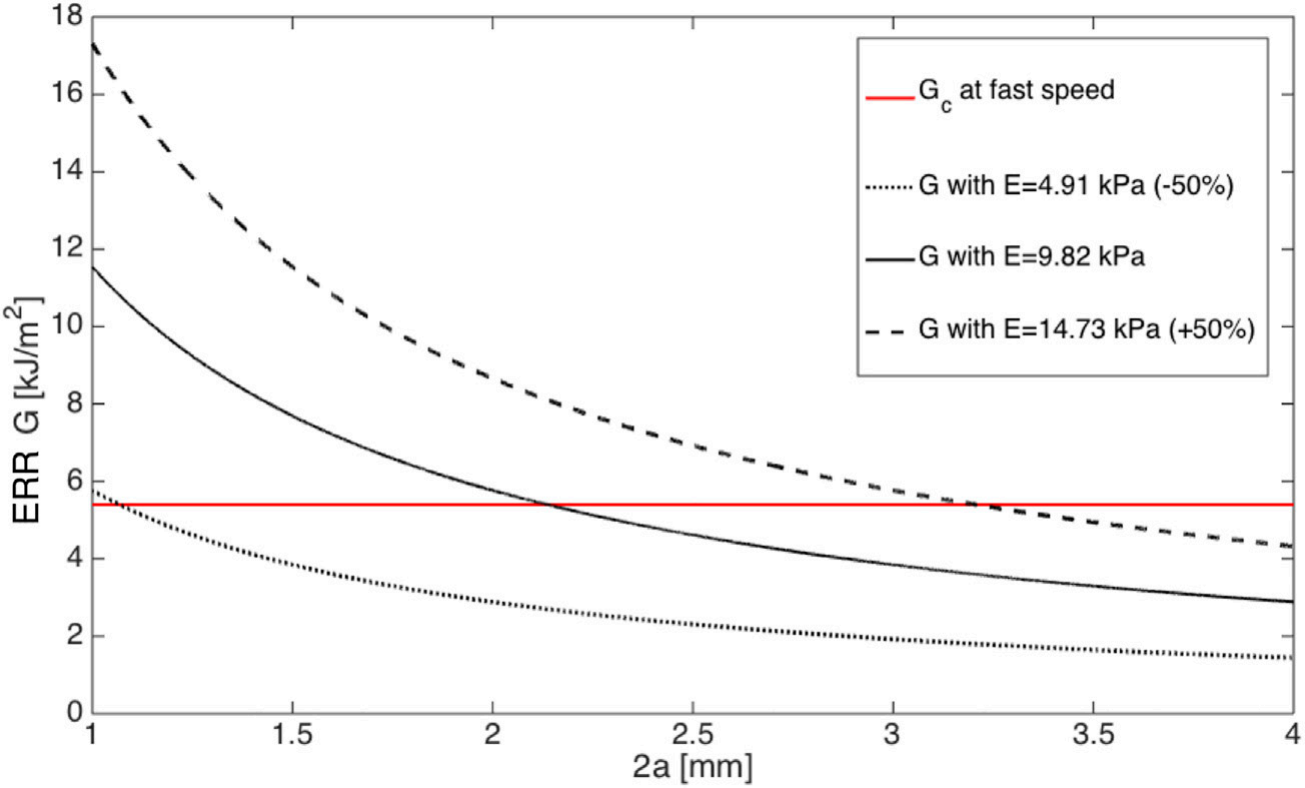
ERRs plotted against the incision length 2*a* in ±50*%* moduli at fast incision speed. Note that *G*_*c*_ is plotted as a reference.

Taken an average injector size of *d* = 1.730 mm amongst the six mentioned systems, the modulus of cornea tissue was shifted by ± 50*%*, then the critical incision length at fast speed changed according to Figure 4, which clearly revealed that ERR with softer material first joined critical ERR and resulted in a smaller minimal incision length.

### 3.2 Numerical Results

**FIGURE 5:**
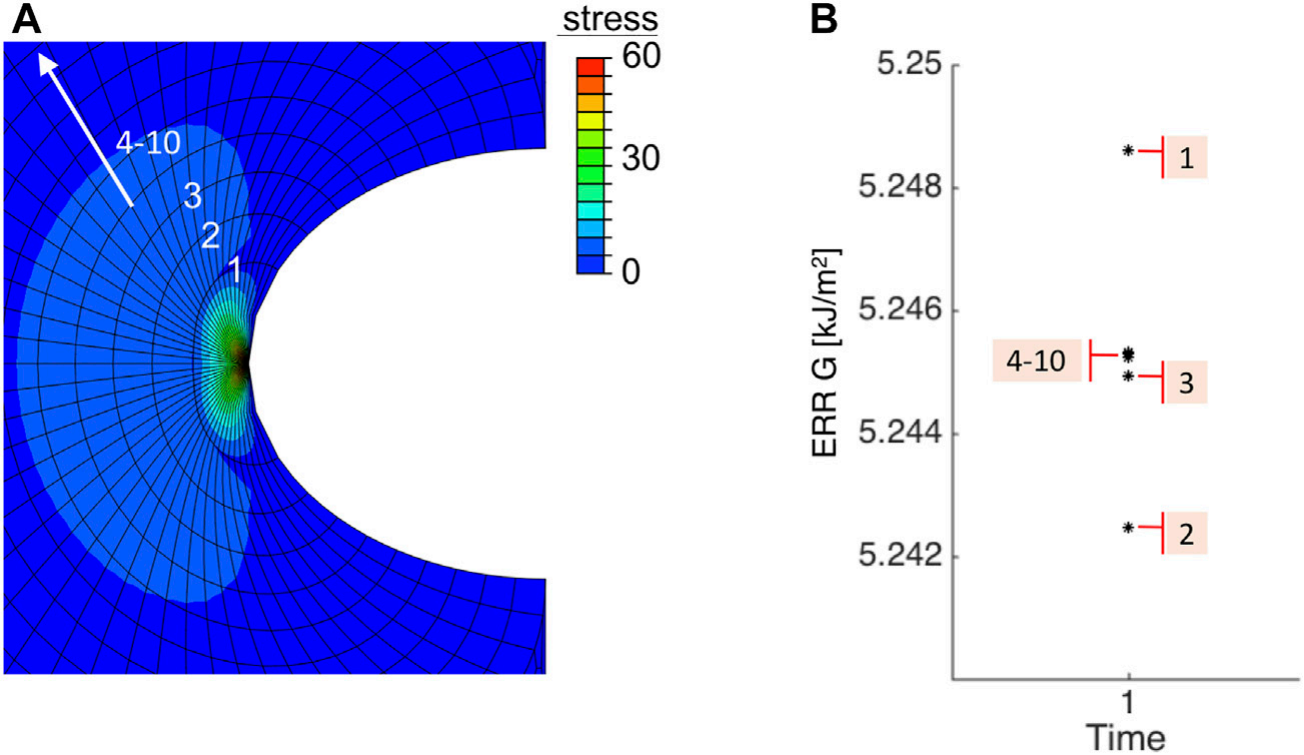
Numerical result of **(A)** the maximal principal in-plane stress and **(B)** values of ERR under 10 contours near the crack tip. Note that the first three contour integrals were neglected.

We then compared our analytical results with numerical simulations. A typical FE result is shown in Figure 5, where the incision length was 2*a* = 2.2 mm, injector size was the average value of six mentioned systems *d* = 1.730 mm and *E* = 9.82 MPa at fast speed, the crack opened subjected to the applied loading, and maximal principal stress was concentrated at the crack tip. For each contour near the crack tip, one corresponding ERR could be output in Abaqus and depicted in Figure 5B. The first three contour integrals are commonly neglected, because the crack tip is so close and can lead to unwanted effects. Therefore, contour integrals 4–10, were used for evaluation and they well converged to a single value of 5.244 *kJ*/*m* ^2^, which agreed with the analytical result 5.246 *kJ*/*m* ^2^ calculated from Eq. 6. Regardless of inducing further damage, ERRs could be calculated within linear theory, however, the tear further propagation, if exists, was not presented in FE simulations.

**FIGURE 6:**
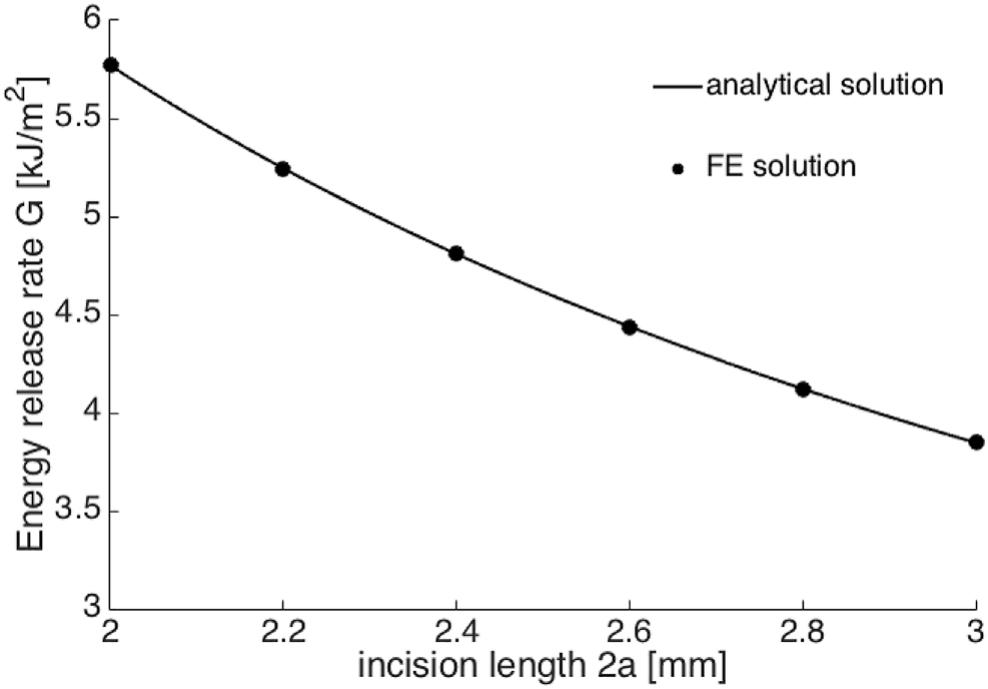
The comparison between FE numerical solutions (black dots) and analytical solutions (black line).

This FE model was then saved as a *Python* script to create new Abaqus jobs. Linux commands were used to edit the incision length 2*a* from 2.0 to 3.0 mm in an increment of 0.2 mm in script. With each 2*a*, corresponding applied stress *σ*_0_ that results in *b*/2 displacement at the middle of crack surface was found by walking around the analytical solutions from Supplementary Equation S8 (the detailed derivation could be found in Supplementary Material). Once matched, the corresponding ERR could be output, which is illustrated in Figure 6. The agreement between the FE simulation and analytical solutions indicated that more realistic scenarios, for example, a circular shape and/or anisotropic corneal material could be further considered and implemented in numerical simulations.

## 4 Discussion

In this study, both the analytical and numerical methods revealed the potential of suggesting there exists a safe and effective surgery incision length for IOL delivery. Taken six commercially available IOL injection systems as examples, the recommended 2.2 mm incision can not be treated as a universal standard.

### 4.1 Effect of Injector Size

The critical incision length is dependent on many factors, a typical one is the size of injector, a larger injector results in a larger required critical pre-existing length. It is also recommended to twist the injector so that the major axis keeps align with the tear during surgery, i.e., the tear opening can be maintained in its smallest magnitude. Studies have shown that stretching this incision or wound enlargement is affected by the type of injector cartridge or insertion method used (Ouchi, 2012). Whereas creating smaller diameter injector nozzles would hamper the ease of IOL insertion. Reducing the thickness of the nozzle material or designing a slit in the nozzle to buffer the stretch force (as seen in a few of IOL designs) would be a good compromise (Nanavaty and Kubrak-Kisza, 2017).

### 4.2 Effect of Insertion Speed

Due to the critical ERR difference in the effect of speeds of IOL insertion on the clear corneal wound structure, fast insertion was better than slow insersion since smaller safe tear length was needed. Other experimental studies also found that slow IOL insertion may affect clear corneal wound structure more than fast IOL insertion, and this appeared to be due to vertical stretching of the insertion cartridge by the IOL (Ouchi, 2012; El Massry et al., 2016). In their experiment, 80 randomized eyes underwent clear corneal phacoemulsification to either fast IOL insertion (one revolution per second) or slow IOL insertion (one-quarter revolution per second). A screw plunger-type injector was used in both groups. The change in wound size after IOL insertion was significantly larger in the slow group. More eyes in the slow group required corneal hydration, and wound structure changes on optical coherence tomography were more prominent.

### 4.3 Effect of Energy Release Rate

The key work in FE simulations was to work out the value of ERR. There are many numerical methods existing for calculating G (Zehnder, 2012), and most rely, not on evaluating the singular stress field at the tips, but rather on the global energy and work, so an accurate value for G can be obtained with modest mesh refinement.

The evaluated *G*_*c*_ gave a floor barrier for safe delivery. We noted that the value of *G*_*c*_ was patient-specific, and in practice could be higher or lower than the values we used here, especially when cornea is in infection or under drugs. If *G*_*c*_ is increased, then the required length is decreased according to Eq. 6, the less likely the tissue is torn.

### 4.4 Effect of Cornea Geometry

The cornea geometry was considered as an infinite 2D plate compared to its central tear. However, in ductile bio-materials like cornea, the energy that rupturing the chemical bonds along the crack plane plays a relatively small role in resisting crack growth, with a large part of fracture energy being associated with plastic flow near the crack tip. This “plastic zone” size should not be so large as to interact with the specimen’s free boundaries or to destroy the basic nature of the singular stress distribution. Hence, due to the finite dimension of cornea, the value of *G*_*c*_ should be modified by a ratio *β* which depends on the geometry of the cornea tissue and location of the pre-existing tear. For example, in a square specimen with a central tear, *β* = 1.12 (Murakami, 1987). The natural curvature and varying thickness of a three-dimensional cornea (Pandolfi, 2020) will need to be carefully treated in further studies.

### 4.5 Effect of Material Property

Figure 4 indicated that having a softer material was optimal to avoid further tissue damage to secure a more successful IOL delivery. However, the quantitative effect of this action on critical ERR is unknown and needs to be further studied.

**FIGURE 7:**
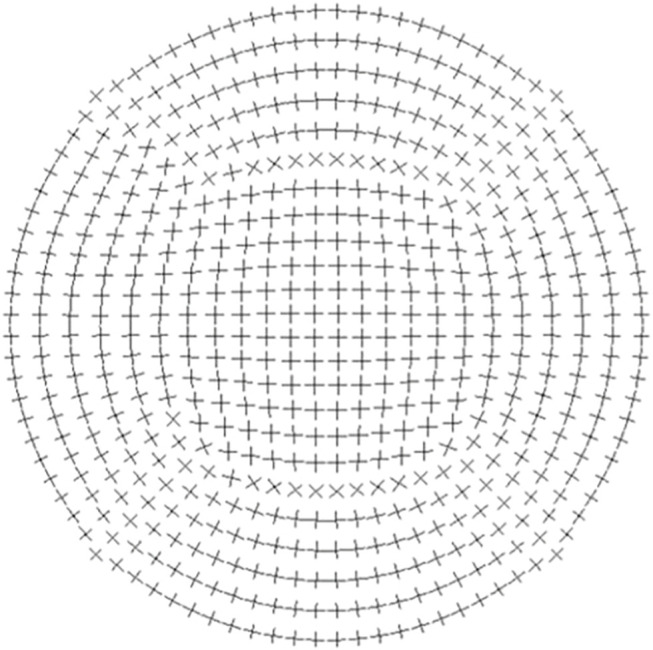
Sketch of fibril orientation adopted from (Pandolfi, 2020). The cornea is composed of bundles of collagen fibrils oriented in orthotropic manner.

The numerical model also allowed us to study a more realistic cornea material. According to X-ray scattering measurements, the layer-wise cornea is characterized by a strong fibril orientation, about 66% of the fibrils are oriented in the 45° sectors along the vertical and the horizontal direction. At the center of the cornea collagen is organized in an orthogonal configuration, and at the limbus it runs circumferentially, as seen in Figure 7. The tissue shows a typical organization of a material with symmetries (Pandolfi and Manganiello, 2006). In this context, it is worth investigating the effect of material organization on ERR. Hence a transversely isotropic material was assigned in the numerical simulations, typically with moduli *E*_*x*_/*E*_*y*_ = *η* and *η* = 0.25, 0.5, 1, 2, 4, respectively, where *E*_*x*_ is the modulus along the tear length and *E*_*y*_ is the one that perpendicular to *E*_*x*_. Table 4 clearly illustrates that *E*_*y*_ is the dominate factor that has effects on ERR, indicating that the incision is preferably along the direction with larger modulus. In practice, it is recommended to conduct a circumferential cut near the limbus.

**TABLE 4:** ERR against the material orthotropy ratio *η*.

*η*	*E*_*x*_ (*MPa*)	*E*_*y*_ (*MPa*)	ERR (*kJ*/*m* ^2^)
2	19.64	9.82	5.301
1.5	14.73	9.82	5.267
1	9.82	9.82	5.244
0.75	9.82	14.73	6.740
0.5	9.82	19.64	8.029

However, the organization of the corneal tissue is far complex than linearity, presenting a clear anisotropic behaviour due to a hierarchically organized collagen, the level of anisotropy is highly location dependent (Bryant and McDonnell, 1996; Daxer and Fratzl, 1997). Several nonlinear constitutive models based on hyperelastic material assumptions have been proposed and developed to represent in various numerical applications, for example, in refractive surgery, tonometer indentation test and air puff test (Simonini and Pandolfi, 2015; Montanino et al., 2018; Pandolfi, 2020). For clinical applications, patient-specific geometrical and material features are required in future FE models.

### 4.6 Limitations

All our estimations were based on model assumptions and the parameters estimated from pigs. This could only serve as a guideline as a more accurate estimation was not possible unless we have access to the material properties of each individual patient.

The elastic modulus of human corneas (Bryant and McDonnell, 1996) of average value of 0.79–0.83 MPa is at least one order smaller compared to the porcine parameters of around 10 MPa used herein. This may explain the reason why the recommended critical incision lengths for different injectors were mostly larger than expected. The trend and the affecting factors listed in Table 2 can serve as a useful guidance for human operations.

## 5 Conclusion

In this work, based on the Griffith’s critical ERR criterion, analytical and numerical solutions were both provided to answer the clinical question, i.e., if the critical incision length is enough for a safe injection in a cataract surgery. Several important factors were examined (e.g., the size of nozzle inserted into the wound, corneal tissue properties and injection speed) quantitatively, indicating that a smaller injection size, a faster insertion speed and a softer material, a larger ERR would guarantee a safer operation.

Our study suggested that the current recommended incision depth of 2.2 mm in IOL delivery was, in most circumstances, not large enough to avoid tearing and tissue damage, as observed in clinical practice. Setting a universal length aiming at all type of injectors was not appropriate and the suggested minimum incision length for different injector diameter is listed in Table 2. Our results supported and explained many clinical findings, suggesting that a surgeon could insert the IOL quickly to lessen wound damage. However, caution must be used with the quick-insertion method because rapid insertion might induce tissue injury, especially with a wound-assisted method. It was also recommended to advance IOL injector *via* its minor axis, and the tear was preferably along the circumferential direction due to tissue orthotropy.

## Data Availability

The original contributions presented in the study are included in the article/Supplementary Material, further inquiries can be directed to the corresponding author.

## Footnotes

1 This size is insignificant to results and was chosen to be large enough compared to tear dimension.
