# Supplementary material for: Estimations of Critical Clear Corneal Incisions Required for Lens Insertion in Cataract Surgery: A Mathematical Aspect
Source: Front Physiol. 2022 Apr 8;13:834214. doi: 10.3389/fphys.2022.834214 (PMC9023857; doi:10.3389/fphys.2022.834214)
Supplement: Supplementary file 1 [file DataSheet1.PDF]

Supplementary Material

1 ANALYTICAL DERIVATION OF DISPLACEMENT AT CRACK SURFACE

For brittle materials exhibiting linear elastic behavior, analytical methods of elasticity are used to obtain stresses and displacements in cracked bodies, such as complex potential function method. In the complex potential method introduced by Kolosov and Muskhelishvili(Sun et al., 2012; Wei, 2010), stresses and displacements in two-dimensional crack problems are expressed in terms of analytic functions of complex variables. The problem of obtaining stresses and displacements around a crack tip is converted to finding some analytic functions subjected to appropriate boundary conditions. Following the general Kolosov-Muskhelishvili formulas, Westerhaard (Westergaard, 1933; Sun et al., 2012) established a more convenient method to compute the basic crack problems.

Let us focus on Mode I crack in plane stress problem, the concept of Airy stress function ϕ is firstly introduced through

$$\sigma_{xx} = \frac{\partial^2\phi}{\partial y^2}, \sigma_{xy} = -\frac{\partial^2\phi}{\partial x \partial y}, \sigma_{yy} = \frac{\partial^2\phi}{\partial x^2} \tag{S1}$$

so that the equilibrium conditions

$$\nabla^2(\sigma_{xx} + \sigma_{yy}) = 0$$

can be automatically satisfied and becomes

$$\nabla^4\phi = \nabla^2\nabla^2\phi = 0 \tag{S2}$$

where ∇^4 is the biharmonic operator.

By representing ϕ in complex potential, the Kolosov-Muskhelishvili formulas express stresses $\sigma_{xx}, \sigma_{xy}\sigma_{yy}$ and displacements u_x, u_y as

$$\sigma_{xx} + \sigma_{yy} = 4Re[\psi'(z)] \tag{S3}$$

$$\sigma_{yy} - \sigma_{xx} + 2i\sigma_{xy} = 2[\overline{z}\psi''(z) + \chi''(z)] \tag{S4}$$

$$2\mu(u_x + iu_y) = \kappa\psi(z) - z\overline{\psi'(z)} - \overline{\chi'(z)} \tag{S5}$$

where $\psi(z)$ and $\chi(z)$ with regard to the complex variable $z = x+iy$ are analytical functions relating to ϕ. The shear modulus $\mu = \frac{E}{2(1+\nu)}$ and $\kappa = (3-\nu)/(1+\nu)$ in a plane stress problem. E and ν are the Young's modulus and the Poisson's ratio of cornea, respectively.

Consider the Mode I problem discussed in this work that an infinite plane with crack along the x-axis, if external loads are symmetric with respect to the x-axis, then $\sigma_{xy} = 0$ along $y = 0$. This important information leads to a correlation between functions $\psi(z)$ and $\chi(z)$ that

$$\overline{z}\psi''(z) + \chi''(z) + A = 0$$

in which A is a real constant. It is thus possible to rewrite the expressions of stresses and displacements in Eqs. S3 through S5 by elaborately design one single function Z_I, via $\psi' = \frac{1}{2}(Z_I + A)$.

Thus $\psi = \frac{1}{2}(\hat{Z}_I + Az)$, where $\hat{Z}_I' = Z_I$. And Z_I is the so-called Westergaard function for Mode I problems.

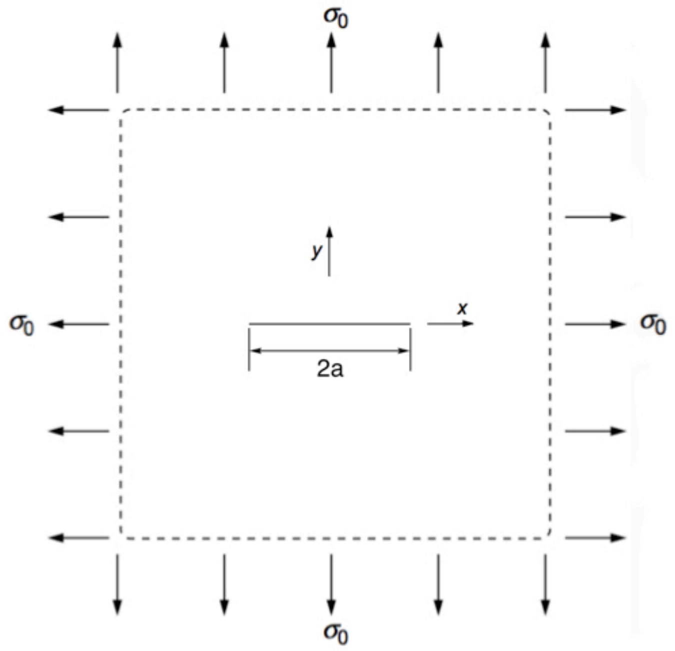

Figure S1. A crack in an infinite plane subjected to biaxial tension σ_0.

For a typical crack problem shown in Fig. S1 that a 2D plane with a line crack of length $2a$ is subjected to biaxial stress σ_0 at infinity. The Westergaard function is given by

$$Z_I = \frac{\sigma_0 z}{\sqrt{z^2 - a^2}}, A = 0. \tag{S6}$$

And the corresponding vertical displacement of the upper crack surface is derived as

$$4\mu u_y = (\kappa + 1) Im\hat{Z}_I = (\kappa + 1)\frac{K_I}{\sqrt{\pi a}}\sqrt{a^2 - x^2}; \tag{S7}$$

where $K_I = \sigma_0\sqrt{\pi a}$ is the intensity factor. Therefore, at the centre of the crack, Eq. S7 reduces to

$$u_{y0} = \frac{\kappa + 1}{4\mu}\sigma_0 a. \tag{S8}$$

Note that the same procedures are carried out for Mode I crack with other boundary conditions, ending in the expressions given in Eq. S7, the only difference being the expressions of K_I.

REFERENCES

Sun, C. T., Jin, Z. H., and Sun, C. T. (2012). Fracture Mechanics (Boston: Academic Press)

Wei, R. P. (2010). Fracture Mechanics: Integration of Mechanics, Materials Science and Chemistry (Cambridge University Press)

Westergaard, H. M. (1933). Stresses at a crack, size of the crack, and the bending of reinforced concrete (Journal Proceedings)
